# LI-RADS: concordance between energy-integrating computed tomography, photon-counting detector computed tomography and magnetic resonance imaging

**DOI:** 10.1186/s40644-025-00922-9

**Published:** 2025-08-14

**Authors:** Lukas Müller, Tobias Jorg, Fabian Stoehr, Jan-Peter Grunz, Dirk Graafen, Moritz C. Halfmann, Henner Huflage, Friedrich Foerster, Jens Mittler, Daniel Pinto dos Santos, Tobias Bäuerle, Roman Kloeckner, Tilman Emrich

**Affiliations:** 1 Department of Diagnostic and Interventional Radiology, University Medical Center Mainz, Langenbeckstr. 1, Mainz, 55131 Germany; 2 Department of Radiology, University of Wisconsin, Madison, WI USA; 3 Department of Diagnostic and Interventional Radiology, University Hospital Würzburg, Würzburg, Germany; 4 Department of Internal Medicine I, University Medical Center Mainz, Mainz, Germany; 5 Department of General, Visceral and Transplant Surgery, University Medical Center Mainz, Mainz, Germany; 6 Institute of Interventional Radiology, University Hospital of Schleswig-Holstein - Campus Lübeck, Lübeck, Germany

**Keywords:** Hepatocellular Carcinoma, Liver Imaging Reporting & Data System, Photon-Counting Computed Tomography, Magnetic Resonance Imaging, Accuracy

## Abstract

**Background:**

Photon-counting detector CT (PCD-CT) offers technical advantages over energy-integrating detector CT (EID-CT) for liver imaging. However, it is unclear whether these translate into clinical improvements regarding the classification of suspicious liver lesions using the Liver Imaging Reporting and Data System (LI-RADS). This study compared the intra- and intermodal agreement of EID-CT and PCD-CT with Magnetic resonance imaging (MRI) for liver lesion classification.

**Methods:**

This retrospective study included patients who underwent EID-CT or PCD-CT and MRI within 30 days between 02/2023 and 01/2024. Three board-certified radiologists assessed LI-RADS classification and presence of LI-RADS major features. Fleiss’ Kappa and intraclass correlation coefficients (ICC) were used to evaluate rater agreement.

**Results:**

Sixty-eight lesions in 26 patients (mean age 65.0 ± 14.2 years, 19 [73.1%] male) were analyzed. Intramodal inter-rater agreement for LI-RADS classification was 0.88 (0.62–0.88) for EID-CT, 0.90 (0.83–0.94) for PCD-CT, and 0.87 (0.81–0.91) for MRI. Agreement in PCD-CT was substantial for all LI-RADS major features, whereas in EID-CT only for washout. Intermodal agreement between CT and MRI ranged from 0.67 to 0.72. Final intermodal LI-RADS classification agreement was higher for PCD-CT (0.72–0.85) than EID-CT (0.52–0.64).

**Conclusions:**

PCD-CT demonstrated higher intermodal and intramodal agreement for LI-RADS classification and major features than EID-CT. Additionally, PCD-CT shows significantly higher intramodal and inter-rater agreement for LI-RADS classification and greater concordance with MRI compared to EID-CT, reaching substantial to almost perfect agreement. These results suggest a potential benefit of PCD-CT in the management and treatment decision-making of HCC.

**Supplementary Information:**

The online version contains supplementary material available at 10.1186/s40644-025-00922-9.

## Background

Chronic liver disease (CLD) is a prevalent and growing global health concern, with rates expected to increase significantly in the coming years [1, 2]. Patients with CLD are at an increased risk of developing hepatocellular carcinoma (HCC), the most common primary liver malignancy [3]. HCC ranks as the sixth most common cancer globally and is the third leading cause of cancer-related mortality worldwide [4]. Noninvasive diagnosis of HCC is the de facto standard of care. According to the guidelines of the European Association for the Study of the Liver and the American Association for the Study of Liver Diseases, computed tomography (CT) and MRI are the mainstay of non-invasive diagnostics [5–7].

In clinical practice, however, high-risk patients frequently present with various focal liver lesions beyond HCC [8]. These lesions range from clearly benign, such as cysts and hemangiomas, to regenerative and dysplastic nodules, as well as malignant tumors such as intrahepatic cholangiocarcinoma, combined hepatocellular-cholangiocarcinoma, and liver metastases [8–10]. To standardize the classification of these diverse findings and improve diagnostic accuracy, the Liver Imaging Reporting and Data System (LI-RADS) offers a structured algorithm designed to categorize liver observations in patients with CLD according to their likelihood of being HCC [11].

In recent years, discrepancies in the diagnostic classification of liver lesions using CT and MRI have been widely reported. Despite moderate to substantial intermodal agreement, diagnoses are marked by considerable variability [8, 12–17]. These differences are mainly attributed to the lower accuracy of CT in assessing LI-RADS major features compared to MRI, which directly impacts LI-RADS categorization and subsequent clinical decision-making [13]. Consequently, many authors advocate for the use of complementary imaging to overcome these limitations; however, a recent study by Basha et al. reported only a modest improvement in accuracy [8, 16, 18].

Currently, all those considerations miss the potential of photon-counting detector CT (PCD-CT), which has not been evaluated for the classification of liver lesions in CLD. While PCD-CT offers several advantages over conventional energy-integrating detector CT (EID-CT) – including superior dose efficiency, spatial resolution, contrast-to-noise ratio, and reduced image noise – it also enables spectral imaging capabilities in every scan, which facilitate virtual monoenergetic imaging (VMI) and iodine quantification [19–22]. While the depiction of LI-RADS features using PCD-CT remains largely unexplored, with current studies primarily focusing on optimizing technical parameters [21, 23, 24], for dual-energy CT techniques it has already been demonstrated that spectral imaging – particularly VMI at low kiloelectronvolt (keV) – can enhance the visualization of HCC features such as arterial hypervascularity, capsule enhancement, and washout [25, 26]. We therefore hypothesized that LI-RADS features can be visualized with high concordance to MRI by combining spectral imaging with the improved spatial resolution offered by PCD-CT.

Therefore, the aim of this study was to evaluate the intra- and intermodal agreement of EID-CT and PCD-CT with MRI in classifying liver lesions, providing insights into the potential of PCD-CT to enhance lesion differentiation and improve LI-RADS classification.

## Methods

This retrospective study was approved by the local ethics committee of Rhineland-Palatinate. Informed consent was waived by the ethics committee (Permit. No. 2022–16359).

### Patient selection

The local radiology information system records were reviewed to identify all patients with a history of CLD who underwent both abdominal CT with a liver protocol and MRI with extracellular contrast agents within a 30-day interval between February 1, 2023, and January 31, 2024. Patients were included according to the following criteria: 1) Suitable for the application of the LI-RADS criteria according to the recommendations of LI-RADS v2018; 2) The observed lesion falls within the LI-RADS category 3 to 5 [11]; and 3) did not undergo locoregional (e.g. ablation, TACE) or systemic treatment; and 4) adequate image quality. All patients fulfilling the inclusion criteria were prepared (patients selection and image work-up) by a radiology resident with 5 years of experience in liver imaging (L.M.) under the supervision of experienced board-certified radiologists (R.K.) with more than 15 years of experience in abdominal imaging to be later identified during the image analysis.

### Image acquisition

EID-CT scans were acquired on a 256-slice CT scanner (Brilliance iCT 256®, Philips Healthcare, Eindhoven, Netherlands), while PCD-CT scans were performed on the first-generation clinical PCD-CT (Naeotom Alpha®, Siemens Healthineers, Erlangen, Germany). EID-CT scans utilized a tube potential of 100 kVp, whereas PCD-CT scans used a 120 kVp tube potential in single-source, multi-energy scan mode (QuantumPlus®, Siemens Healthineers). All images were reconstructed with slice thicknesses of 1 mm and 3 mm. Furthermore, for PCD-CT all images were reconstructed as VMIs at 50 keV, which was reported to be the optimum keV level for abdominal PCD-CT in a previous study [22]. Detailed acquisition parameters have been reported in previous publications [22–24]. Identical weight-adjusted contrast media protocols were applied for both CT types: for patients < 70 kg, 80 ml of iodine contrast agent at 3.5 ml/s; for 70–90 kg, 100 ml at 4 ml/s; and for > 90 kg, 120 ml at 4 ml/s (Ultravist® 370, Bayer Vital, Leverkusen, Germany), resulting in iodine fluxes of 1.3 gI/s and 1.5 gI/s, respectively. Each contrast injection was followed by a 50 ml saline bolus at 3 ml/s. The late arterial phase was triggered by bolus tracking in the proximal abdominal aorta, with a threshold increase of 100 HU and a post-threshold delay of 13 s. Portal venous and delayed phases were acquired with delays of 50 s and 180 s, respectively [24].

MRI examinations were performed using various scanners: 1.5 T Sonata®, 1.5 T Avanto®, 3 T Vida®, 3 T Prisma®, or 3 T Skyra® (all from Siemens Healthineers, Erlangen, Germany). The standardized MRI protocol included single-breath-hold and respiratory-triggered T2-weighted 2D turbo spin-echo sequences, diffusion-weighted imaging (DWI), T1-weighted 2D dual gradient-recalled echo (GRE) sequences (in-phase and opposed-phase), and T1-weighted 3D GRE sequences with fat suppression. Images were acquired both before and after contrast agent administration (four dynamic phases): The arterial, portal venous, equilibrium, and delayed phases were initiated at 20, 45, 90, and 150–180 s, respectively, following contrast administration via a power injector [27]. The used contrast agent was Clariscan® (gadoterate meglumine; GE Healthcare Buchler GmbH & Co. KG, Munich Germany).

### Image analysis

**Fig. 1:**
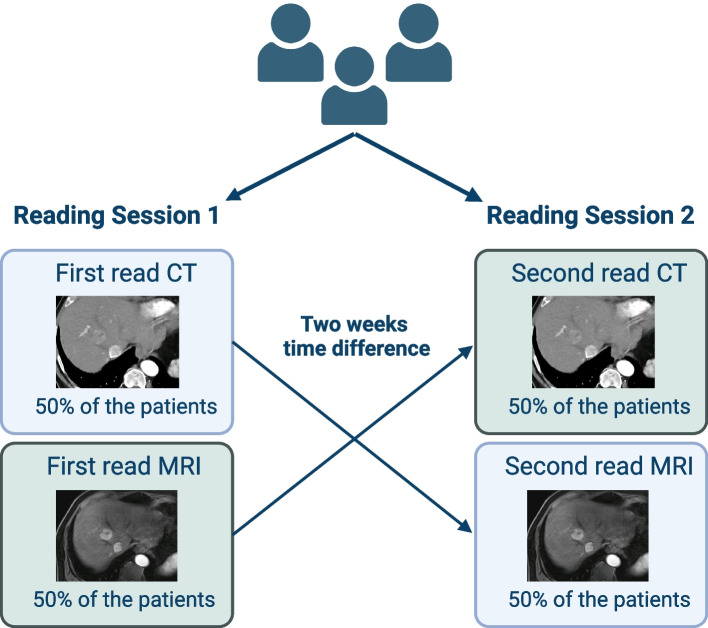
Sequential reading of CT and MRI scans across two sessions

Images were independently analyzed by three board-certified radiologists with experience in abdominal imaging between 6 and 7 years (T.J., J.-P.G., F.S.) using the institution’s picture archiving and communication system (Sectra, Linköping, Sweden). Two reading sessions were conducted, spaced two weeks apart. In the first session, each patient had only one imaging study reviewed: a CT scan for half of the patients and an MRI scan for the other half. In the second session, the alternate imaging study was reviewed for each patient (Fig. 1).

**Fig. 2:**
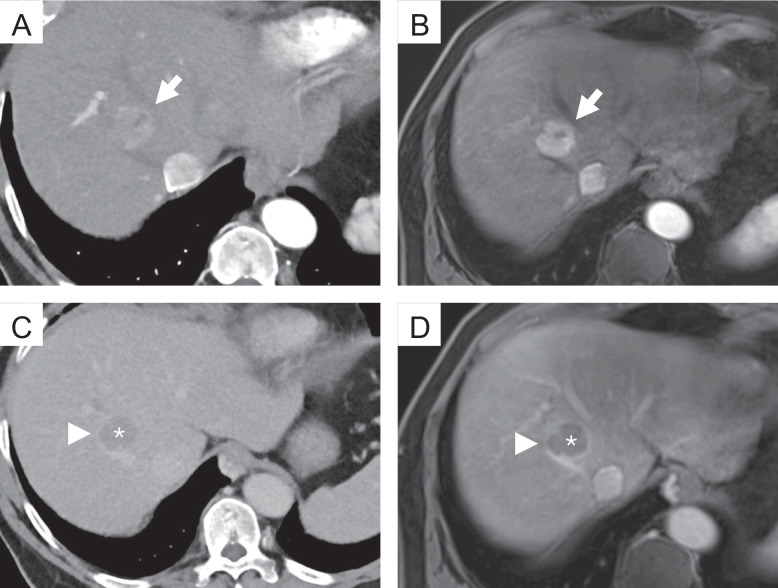
Examples of the different major features of the LI-RADS classification. Top row: Photon-counting detector computed tomography (PCD-CT) (**A**) and magnetic resonance imaging (MRI) (**B**), hepatic arterial phase; the lesion shows strong contrast enhancement compared to the surrounding liver parenchyma (non-rim arterial phase hyperenhancement, APHE) (arrow). Bottom row: PCD-CT (**C**) and MRI (**D**), delayed phase; the lesion shows a reduction in enhancement relative to the surrounding background liver (non-peripheral “washout”) (*). Note the smooth border around the largest parts of the observation (enhancing “capsule” (arrowhead))

During the case review, the raters were provided with the selected studies in hepatic arterial, portal venous, and delayed phase for the specified modality. The raters were allowed to scroll through the images and adjust the display's window and level settings. However, they had no access to additional imaging studies or the patient's clinical information. For each lesion, the three raters documented the lesion size, noted the presence or absence of major features (non-rim arterial phase hyperenhancement (APHE), non-peripheral “washout”, enhancing “capsule”, see examples in Fig. 2), and assigned a LI-RADS category following the LI-RADS v2018 algorithm and diagnostic table [11]. No additional training was required prior to these readings, as the routine application of LI-RADS is well-established among the raters. Examples for the different major features can be found in Fig. 2.

### Statistics

All statistical analyses and graphic outputs were performed with dedicated statistical software (R, version 4.1.1, R Foundation for Statistical Computing, Vienna, Austria). Nominal-scaled variables are reported as absolute numbers and percentages and ordinal-scaled variables as medians and interquartile ranges (IQRs). Interval-scaled variables are reported as means and standard deviations (SDs). Shapiro–Wilk-Test was used to test for normal distribution. Non-normal distributed continuous variables were compared using Mann–Whitney U test, and normal distributed, continuous variables using independent T-test. Dichotomous characteristics of patients were compared with χ^2^ test. Agreement for continuous variables was calculated using the Intraclass Correlation Coefficient (ICC) based on absolute agreement in a two-way random effects model with the following interpretation: < 0.5, poor; 0.50 – 0.75, moderate; 0.76 – 0.90, good; > 0.90, excellent [28]. Agreement for caterogial variables was calculated using Fleiss Kappa with the following interpretation: < 0.00, poor; 0.00–0.20 slight; 0.21 – 0.40, fair; 0.41 –0.60, moderate; 0.61 – 0.80, substantial; and > 0.80, almost perfect [29]. A *p*-value < 0.05 was considered to indicate statistical significance.

## Results

A total of 68 lesions of 26 patients (mean age 65.0 ± 14.2 [SD] years, 19 (73.1%) male) were included in the final analysis while a total of 5 patients not fulfilling the inclusion criteria were excluded. The median interval between CT and MRI was 19.3 ± 7.5 [SD] days, and CT was performed as the first study in 21 (80.8%) patients. Baseline characteristics of the CT groups are compared in Table 1.

**Table 1:** Baseline characteristics with a comparison of both CT groups

Characteristics	EID-CT group (*n* = 10)	PCD-CT group (*n* = 16)	*P* value
Mean Age (SD), years	59.5 (17.7)	68.4 (10.8)	0.30
Sex, n (%)			> 0.90
Male	7 (70.0)	12 (75.0)	
Female	3 (30.0)	4 (25.0)	
CLD, n (%)			0.29
Alcohol	7 (70.0)	9 (56.3)	
HBV	0	1 (6.3)	
HCV	1 (10.0)	3 (18.8)	
Cryptogenic	0	3 (18.8)	
MASH	1 (10.0)	0	
AIH	1 (10.0)	0	
Mean Time CT to MRI (SD), days	20.1 (6.0)	18.8 (8.5)	0.66
Number of lesions, total	27	41	0.09
Mean number of lesions per patient, mean (SD)	2.6 (1.8)	2.7 (1.4)	0.57
Late arterial phase			
Mean CTDI_vol,_ (SD), mGy	14.4 (0.32)	10.0 (2.6)	< 0.01
Mean DLP (SD), mGy × cm	504 (71)	292 (99)	< 0.01
Mean Effective Dose (SD), mSv	7.6 (1.1)	4.4 (1.5)	< 0.01
Portal venous phase			
Mean CTDI_vol,_ (SD), mGy	15.0 (6.0)	9.8 (2.1)	< 0.01
Mean DLP (SD), mGy × cm	552 (180)	334 (194)	< 0.01
Mean Effective Dose (SD), mSv	8.3 (2.7)	5.0 (2.9)	< 0.01
Delayed phase			
Mean CTDI_vol,_ (SD), mGy	14.6 (5.8)	9.8 (2.3)	< 0.01
Mean DLP (SD), mGy × cm	502 (207)	282 (86)	< 0.01
Mean Effective Dose (SD), mSv	7.8 (3.1)	4.2 (1.3)	< 0.01
Mean BMI (SD), kg/m^2^	26.3 (4.3)	26.3 (3.9)	> 0.90

*Abbreviations*: *EID-CT* Energy-integrating detector computed tomography, *PCD-CT* Photon-counting detector CT, *CLD* Chronic liver disease, *HBV* Hepatitis B virus, *HCV* Hepatitis C virus, *MASH* Metabolic Dysfunction-Associated Steatohepatitis, *AIH* Autoimmune hepatitis

### Intramodal comparison

Overall, the inter-rater agreement for the LI-RADS classification was 0.85 (95%CI 0.79 – 0.90) for CT in general, and 0.87 (95% CI 0.81 – 0.91) for MRI. Table 2 shows the distribution of the given major features and the agreement between the raters within the imaging modality. Except for capsule appearance and lesion size, agreement was higher for MRI.

**Table 2:** Inter-rater agreement for LI-RADS major features

Modality	CT (*n* = 68)			MRI (*n* = 68)		
Feature	Frequency (Rater 1/2/3)	*P* value	Agreement	Frequency (Rater 1/2/3)	*P* value	Agreement
APHE	59/60/61	0.60	0.72 (0.45 – 0.91)	57/55/56	0.55	0.83 (0.67 – 0.95)
PV/DP washout	20/27/24	0.06	0.72 (0.57 – 0.86)	28/27/24	0.24	0.81 (0.68 – 0.92)
Capsule appearance	14/15/16	0.78	0.66 (0.47 – 0.80)	19/17/13	0.12	0.65 (0.47 – 0.80)
Lesion size	13/12/12^a^		0.99 (0.99 – 1.00)	13/13/13^a^		0.99 (0.99 – 1.00)

Fleiss Kappa was used for arterial phase hyperenhancement (APHE), portal venous (PV)/delayed phase (DP) washout, and capsule appearance; intraclass correlation coefficient for lesion size

^a^Values given in mm

For EID-CT the inter-rater agreement for the LI-RADS classification was 0.78 (0.62 – 0.88) compared to 0.90 (0.83 – 0.94) for PCD-CT. Table 3 shows the agreement for the different major features of the different CT techniques.

**Table 3:** Inter-rater agreement for EID-CT and PCD-CT

Feature	EID-CT	PCD-CT
APHE	0.58 (0.13 – 1.00)	0.78 (0.46 – 1.00)
Portal venous or delayed phase washout	0.65 (0.36 – 0.88)	0.76 (0.59 – 0.89)
Capsule appearance	0.45 (0.06 – 0.84)	0.69 (0.47 – 0.86)
Lesion size	0.99 (0.99 – 1.00)	0.99 (0.99 – 1.00)

Fleiss Kappa was used for arterial phase hyperenhancement (APHE), portal venous (PV)/delayed phase (DP) washout, and capsule appearance; intraclass correlation coefficient for lesion size

Overall, agreement was higher for PCD-CT compared to EID-CT, except for lesion size, where both techniques demonstrated almost perfect agreement. Notably, the agreement for APHE and washout appearance with PCD-CT closely approximated that of MRI and was even higher for capsule appearance.

### Intermodal comparison

**Fig. 3:**
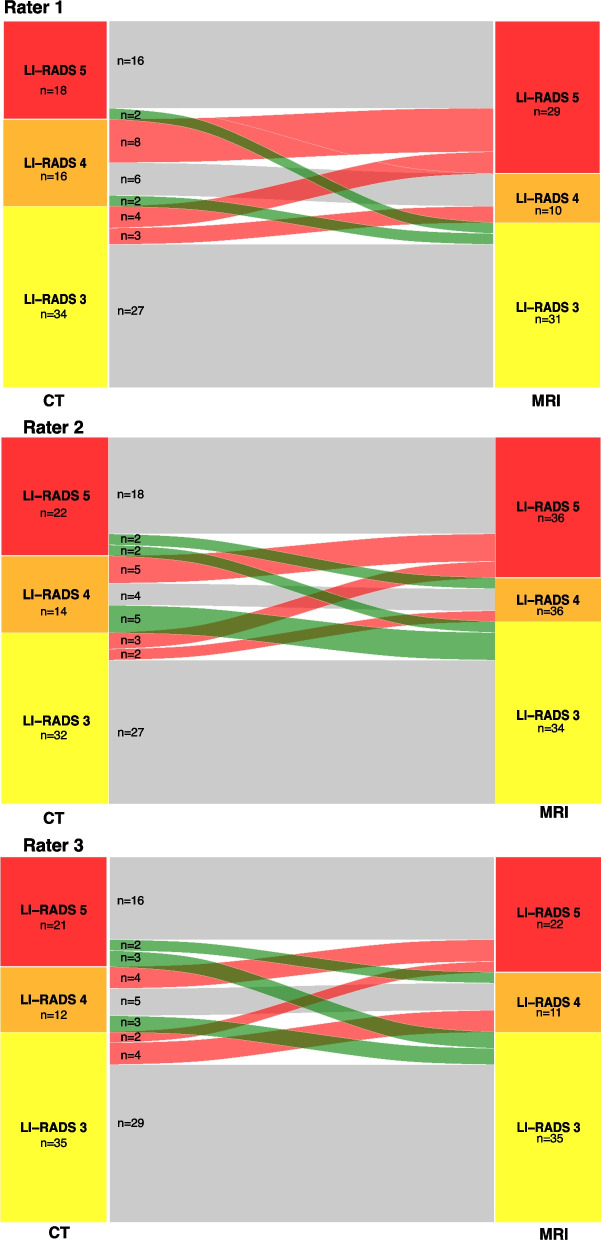
Sankey diagram demonstrating the number of reclassifications from CT to MRI

Overall, the intermodal agreement between CT and MRI ranged between 0.67 and 0.72 for all three raters. The reclassification rates from CT to MRI are depicted in Fig. 3 and in Supplementary Table 1.

For the major features, agreement is depicted in Table 4.

**Table 4:** Intra-rater agreement for CT and MRI

Feature	Rater 1	Rater 2	Rater 3
Portal venous or delayed phase washout	0.55 (0.32 – 0.74)	0.69 (0.51 – 0.85)	0.61 (0.40 – 0.79)
Capsule appearance	0.24 (0.04 – 0.50)	0.43 (0.17 – 0.66)	0.61 (0.34 – 0.82)
Lesion size	0.96 (0.93 – 0.98)	0.96 (0.94 – 0.98)	0.95 (0.92 – 0.97)

Fleiss Kappa was used for portal venous (PV)/delayed phase (DP) washout, and capsule appearance; intraclass correlation coefficient for lesion size. Arterial phase hyperenhancement (APHE) was not investigated as Fleiss Kappa was not applicable due to low number of lesions without arterial enhancement

Table 5 shows the inter-rater agreement for the LI-RADS classification and the major features for EID- and PCD-CT versus MRI, respectively. Notably, the LI-RADS agreement was higher for all raters in the PCD-CT group (0.72 to 0.85) compared to the EID-CT group (0.52 to 0.64).

**Table 5:** Intra-rater agreement for EID-CT and PCD-CT versus MRI

	Rater 1		Rater 2		Rater 3	
	EID-CT	PCD-CT	EID-CT	PCD-CT	EID-CT	PCD-CT
LI-RADS classification	0.60 (0.16 – 0.82)	0.72 (0.53 – 0.84)	0.52 (0.18 – 0.75)	0.85 (0.73 – 0.92)	0.64 (0.36 – 0.82)	0.74 (0.56 – 0.85)
Major features						
Portal venous or delayed phase washout	0.33 (0.11 – 0.69)	0.69 (0.41 – 0.90)	0.43 (0.02 – 0.76)	0.85 (0.66 – 1.00)	0.43 (0.04 – 0.76)	0.73 (0.48 – 0.94)
Capsule appearance	0.03 (-0.31 – 0.35)	0.38 (0.01 – 0.67)	0.20 (-0.22 – 0.66)	0.55 (0.21 – 0.81)	0.18 (-0.17 – 0.65)	0.75 (0.48 – 0.94)
Lesion size	0.93 (0.86 – 0.97)	0.97 (0.95 – 0.99)	0.94 (0.88 – 0.97)	0.98 (0.96 – 0.99)	0.92 (0.81 – 0.97)	0.98 (0.95 – 0.99)

Fleiss Kappa was used for portal venous (PV)/delayed phase (DP) washout, and capsule appearance; intraclass correlation coefficient for lesion size; Arterial phase hyperenhancement (APHE) was not investigated as Fleiss Kappa was not applicable due to low number of lesions without arterial enhancement

**Fig. 4:**
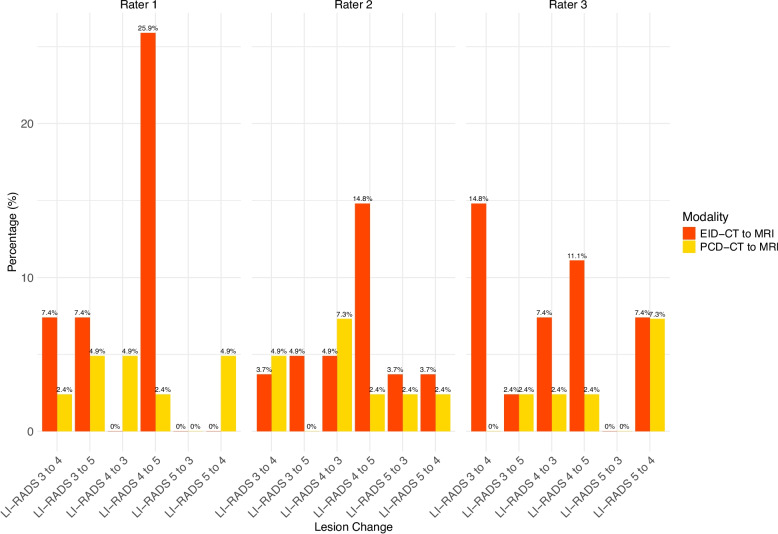
Comparison of reclassification rates from CT to MRI for EID-CT vs PCD-CT

Distribution of the LI-RADS reclassifications can be found in Fig. 4 and Supplementary Table 2.

The rates of reclassification to higher LI-RADS from PCD-CT to MRI were 4/41 (9.8%) for rater 1, 3/41 (7.3%) for rater 2, and 3/41 (7.3%) for rater 3. The rates of reclassification to lower LI-RADS from PCT-CT to MRI LI-RADS were 4/41 (9.8%) for rater 1, 5/41 (12.2%) for rater 2, and 4/41 for rater 3 (9.8%).

The rates of reclassification to higher LI-RADS from EID-CT to MRI were 11/27 (40.7%) for rater 1, 7/27 (25.9%) for rater 2, and 8/27 (29.6%) for rater 3. The rates of reclassification to lower LI-RADS from EID-CT to MRI LI-RADS were 0/27 for rater 1, 4/27 (14.8%) for rater 2, and 4/27 for rater 3 (14.8%).

## Discussion

In this study, we aimed to investigate the concordance between CT and MRI with a special focus on the comparison of EID-CT and PCD-CT. While intramodal inter-rater agreement was slightly higher for MRI compared to CT in general, PCD-CT showed the highest agreement for LI-RADS classification. Besides the higher intramodal inter-rater agreement, PCD-CT also showed a higher intermodal agreement with MRI across all raters (0.72 to 0.85) compared to the EID-CT (0.52 to 0.64). Furthermore, PCD-CT had a remarkably lower number of reclassifications to higher LI-RADS from CT to MRI across all raters.

Several studies have investigated the intramodal inter-rater agreement for EID-CT and MRI before [12–17]. Chernyak et al. compared the intra- and intermodal performance of two raters. In their study, the intramodal agreement for LI-RADS classification was 0.79 and 0.60 for CT and MRI, respectively [12]. While CT agreement was comparable to our results (0.85), MRI agreement was significantly higher in our study (0.87), and therefore more in line with the publication from Basha et al., who report an intramodal inter-rater agreement of 0.93 for MRI and 0.90 for CT [16]. Notably, PCD-CT showed a considerably higher intramodal, inter-rater agreement (0.90) versus EID-CT (0.78) in our study.

For APHE, the intermodal intra-rater agreement has been reported between 0.54 and 0.85 for CT, which is in line with the substantial agreement in our study [12, 14–17]. However, PCD-CT showed a higher agreement (0.78 versus 0.58 in EID-CT). Using MRI, APHE inter-rater agreement (0.83) falls within the reported range of 0.22 to 0.86 [12, 14–17]. In our study, for the washout appearance, intermodal inter-rater agreement was 0.72 using CT and 0.81 using MRI, with remarkable differences between PCD-CT and EID-CT (0.76 versus 0.65). Previous studies have reported an agreement of 0.39 to 0.83, for CT and 0.54 to 0.83 for MRI [12, 14–17]. For the major feature capsule appearance, intramodal inter-rater agreement was noteworthy higher in PCD-CT (0.69 versus 0.45 in EID-CT). Overall, agreement was substantial for both CT and MRI (0.66 and 0.65, respectively), which is in the upper range of previous reports (-0.02 to 0.86 for CT and 0.59 to 0.74 for MRI) [12, 14–17]. The intramodal inter-rater agreement of the lesion size has been almost perfect throughout all modalities in our study, which is in line with previous reports [12, 16, 17].

Previous studies have reported an intermodal agreement of CT and MRI ranging from 0.33 to 0.77 [8, 12, 16]. In our study, the overall intermodal agreement was within this range for all three raters. Notably, for PCD-CT the intermodal agreement ranged from 0.72 to 0.85, and therefore in the upper range or even above the reported values.

In our study, the number of LI-RADS lesions upgraded from CT to MRI ranged between 10 (14.7%) and 17 (25.0%) for all raters and from MRI to CT between 4 (5.9%) and 9 (13.2%). Overall, the number of lesion upgrades seems to be slightly below previous reports. Particularly, for PCD-CT, where the number of lesions upgraded from CT to MRI ranged between 7–10%, compared to 26–41% for EID-CT. Notably, the number of lesions upgraded from MRI to CT ranged from 10–12% in PCD-CT and 0–15% in EID-CT between the raters. Overall, the existing literature regarding the upgrade of LI-RADS lesions between EID-CT and MRI shows a significant variability: Basha et al. report an upgrade from CT to MRI in 22.7% for LI-RADS 3 and 70.8% of LI-RADS 4 lesions, and no upgrade from MRI to CT [16]. Chernyak et al. describe an upgrade from CT to MRI for 10/50 lesions (20.0%) for both raters and from MRI to CT for 12 and 14/50 lesions (24% and 28%, respectively) [12]. Corwin et al. reported a significant upgrade from CT to MRI for 42.5% of overall observations [13]. Looking into detail, in their study, 6 of 42 (14.3%) LI-RADS 3 lesions were upgraded and 8 of 19 (42.1%) LI-RADS 4 lesions. In addition, 2 of 84 (2.4%) LI-RADS 3 lesions were upgraded from MRI to CT, slightly lower than our multileader results, which could be attributed to the performed consensus reading leading to more certainty. Hope et al. reported an upgrade of 10 lesions from LI-RADS 3 to LI-RADS 4 from CT to MRI (around 45%) and 5 lesions from LI-RADS 4 to LI-RADS 5 (around 39%) (note: percentage estimated from the presented bar plots and corresponding legends in the Fig. 1 in [15] as the article does not offer the absolute numbers). A recent study by Agnello et al. reports a higher categorization in MRI for 22/26 (77%) of their lesions for MRI with extracellular contrast agent and 11/40 (28%) of their lesions for MRI with hepatobiliary contrast agent [8]. Overall, PCD-CT shows a remarkable low number of reclassifications.

Regarding the different image features, intermodal agreement for APHE was poor in our study, which might be attributable to the small number of observations without APHE [14]. However, the range of intermodal agreement has been highly variable in previous studies ranging from 0.20 to 0.62 [12, 16, 17]. Furthermore, APHE for PCD-CT and MRI ranged from 0.14 to 0.36, and was therefore somehow comparable with these results. For washout appearance these previous studies have reported a range of intermodal agreement from 0.38 to 0.93. In our study, agreement for this feature ranged from 0.55 to 0.69, with higher agreement in the PCD-CT group (0.69 to 0.85 versus 0.33 to 0.43 in EID-CT). Capsule appearance has shown a high variability in previous studies, which was confirmed in our study with high variability between the three raters [12, 16, 17]. Noteworthy, PCD-CT outperformed EID-CT in the agreement to MRI in this major feature.

Prior studies have shown that dual-energy CT-based spectral imaging can significantly improve the depiction of key LI-RADS features. Reimer et al. demonstrated that 40 keV VMI from spectral detector CT significantly improved washout conspicuity through increased lesion contrast, with quantitative gains in both contrast-to-noise ratio and washout amplitude [25]. Voss et al. showed that 50 keV VMI increased reader confidence in detecting arterial hyperenhancement, capsule enhancement, and washout in small HCC lesions [26]. For PCD-CT, Graafen et al. were able to show that low-keV VMI yield significantly improved objective and subjective quality of arterial phase oncological imaging compared with conventional EID-CT [22]. Building on these advances using spectral imaging, PCD-CT adds the benefit of markedly improved spatial resolution due to its direct-conversion detector design. This suggests that the combination of spectral imaging and high spatial resolution in PCD-CT may further enhance the depiction of subtle HCC features such as capsular enhancement and faint washout, especially in small lesions. However, further dedicated studies are needed to confirm these advantages in clinical LI-RADS assessment.

For clinical decision-making, the higher intramodal and intermodal agreement between CT and MRI as well as the upgrades of the LI-RADS classification between the lesions could lead to a higher reliability between the results of both methods, particularly in patients with different imaging modalities used for follow-up during the course of disease or impossible complementary imaging. However, future evaluation of the interchangeability of PCD-CT and MRI in a prospective randomized setting is mandatory. Following such investigations as well as large-scale histopathologic correlation, the role of CT in the management of LI-RADS lesions could be redefined and emphasized in future guidelines. At the moment, complementary imaging is needed in patients with unclear and discordant observations. However, even in these lesions, PCD-CT could lead to a better understanding about when we should use MRI and when we should use CT. In patients with HCC and underlying liver cirrhosis, this consideration is particularly relevant. Beyond common contraindications for MRI (such as pacemakers, other non-MRI-compatible devices, or claustrophobia) patients with liver cirrhosis frequently present with ascites, which can compromise image quality. Additionally, their often reduced general condition may render MRI unfeasible. While MRI remains the preferred imaging modality according to current guidelines, clinical practice highlights a clear need to optimize CT imaging as a practical and effective alternative for this patient population.

PCD-CT combines spectral imaging with significantly improved spatial resolution. The smaller detector pixels and absence of interpixel septa lead to enhanced edge sharpness and detail visibility. This could be particularly relevant for detecting subtle morphologic features such as capsule enhancement and washout, which are essential for accurate LI-RADS classification. Furthermore, PCD-CT reduced electronic noise leads to an improved CNR, which could be particularly beneficial for detecting washout. Moreover, spectral reconstructions using VMI at low-keV improve lesion-to-liver contrast and enhance the detectability of APHE and washout. As mentioned previously, Reimer et al. and Voss et al. showed that such reconstructions in dual-energy CT improve visualization of these key features – benefits that PCD-CT can deliver with even higher spatial fidelity [25, 26]. Together, these advantages likely contribute to the higher intermodal agreement with MRI observed in our study for PCD-CT compared to EID-CT, and explain the lower rate of LI-RADS reclassifications from CT to MRI. The more confident and accurate identification of LI-RADS major features with PCD-CT appears to reduce diagnostic uncertainty and improve alignment with MRI-based LI-RADS classification.

This study has several limitations. First and foremost, the study has a retrospective design and only includes patients from a single center limiting the number of patients. On the one hand this leads to a higher comparability due to the same used image protocols and standards. On the other hand, there could be a variability for other image protocols and different distributions of lesions at other institutions. Thus, in the future we aim to build a registry for LI-RADS classification using PCD-CT at different institutions. Secondly, although we included one external rater to avoid familiarity bias, this cannot be fully excluded for the other raters. Thirdly, histologic work-up was only available for a limited number of patients as the diagnosis of HCC does not require histologic work up and can be based on image criteria according to Western guidelines [5, 6]. The correlation of LI-RADS with histopathologic results has been, however, investigated extensively in previous studies [30–32]. Fourthly, we only included patients with ECA-MRI as the number of EOB-MRI would have been small due to standards at our institution. Although the potential benefits of a hepatobiliary phase for differentiation of LI-RADS lesions has been shown for EOB-MRI, in a recent study, Agnello et al. have shown that ECA-MRI had the highest accuracy for LI-RADS 4 and 5 as predictors for HCC [8]. Fifthly, we offered the raters just the standard reconstructions within the PACS. For PCD-CT this included virtual monoenergetic images at 50 keV with optimized QIR and kernels [22–24]. However, the optimal keV for HCC detection still might differ between different patients, and setting can be adapted for personal preferences using dedicating software. Lastly, there was a difference in the number of observed lesions between PCD-CT plus MRI and EID-CT plus MRI.

## Conclusion

PCD-CT showed a higher intramodal inter-rater agreement as well as higher concordance with MRI compared to EID-CT for the overall classification of LI-RADS lesions, reaching substantial to almost perfect agreement. Furthermore, the intermodal agreement for the major features of the LI-RADS classification was higher compared to EID-CT, with values comparable to MRI. Additionally, the number of lesion upgrades from PCD-CT to MRI was noteworthy lower. Thus, PCD-CT could be beneficial in the management and treatment decision-making in patients with different image modalities used for follow-up during the course of disease or in patients that cannot undergo MRI (e.g. pacemaker, claustrophobia, image quality-impacting presence of ascites, but also bad overall status of the patients). Future studies are mandatory to assess the interchangeability and the potential for omission of redundant imaging.

## Supplementary Information

Supplementary Material 1.

## Data Availability

The datasets presented in this article are not readily available because data cannot be shared publicly because of institutional and national data policy restrictions imposed by the local Ethics. Data are available upon request for researchers who meet the criteria for access to confidential data.

## Abbreviations

APHE: Arterial phase hyperenhancement
CLD: Chronic liver disease
CT: Computed tomography
EID-CT: Energy-integrating detector CT
HCC: Hepatocellular carcinoma
ICC: Intraclass correlation coefficient
IQR: Interquartile range
LI-RADS: Liver Imaging Reporting and Data System
MRI: Magnetic resonance imaging
PCD-CT: Photon-counting detector CT
SD: Standard deviation
VMI: Virtual monoenergetic imaging
