# Supplementary material for: LI-RADS: concordance between energy-integrating computed tomography, photon-counting detector computed tomography and magnetic resonance imaging
Source: Cancer Imaging. 2025 Aug 14;25:99. doi: 10.1186/s40644-025-00922-9 (PMC12351820; doi:10.1186/s40644-025-00922-9)
Supplement: Supplementary file 1 — Supplementary Material 1. [file 40644_2025_922_MOESM1_ESM.docx]

**Supplementary Table 1.** Rater agreement for LI-RADS category assignment between CT and MRI

| Rater 1 (Agreement 0.67 (0.51 – 0.78)) | | MRI | | |
| --- | --- | --- | --- | --- |
|  |  | LI-RADS 3 | LI-RADS 4 | LI-RADS 5 |
| CT | LI-RADS 3 | 27 | 3 | 4 |
|  | LI-RADS 4 | 2 | 6 | 8 |
|  | LI-RADS 5 | 2 | 0 | 16 |
| Rater 2 (Agreement 0.72 (0.59 – 0.82)) | | MRI |  |  |
|  |  | LI-RADS 3 | LI-RADS 4 | LI-RADS 5 |
| CT | LI-RADS 3 | 27 | 3 | 2 |
|  | LI-RADS 4 | 5 | 4 | 5 |
|  | LI-RADS 5 | 2 | 2 | 18 |
| Rater 3 (Agreement 0.70 (0.55 – 0.80)) | | MRI |  |  |
|  |  | LI-RADS 3 | LI-RADS 4 | LI-RADS 5 |
| CT | LI-RADS 3 | 29 | 4 | 2 |
|  | LI-RADS 4 | 3 | 5 | 4 |
|  | LI-RADS 5 | 3 | 2 | 16 |

**Supplementary Table 2.** LI-RADS category changes of the lesions between PCD-CT/EID-CT and MRI

|  | Rater 1 | | Rater 2 | | Rater 3 | |
| --- | --- | --- | --- | --- | --- | --- |
| LIRADS category change | PCD-CT to MRI (n=41) | EID-CT to MRI (n=27) | PCD-CT to MRI (n=41) | EID-CT to MRI (n=27) | PCD-CT to MRI (n=41) | EID-CT to MRI (n=27) |
| LI-RADS 3 to 4 | 1 (2.4%) | 2 (7.4%) | 2 (4.9%) | 1 (3.7%) | 0 | 4 (14.8%) |
| LI-RADS 3 to 5 | 2 (4.9%) | 2 (7.4%) | 0 | 2 (4.9%) | 1 (2.4%) | 1 (2.4%) |
| LI-RADS 4 to 5 | 1 (2.4%) | 7 (25.9%) | 1 (2.4%) | 4 (14.8%) | 1 (2.4%) | 3 (11.1%) |
| LI-RADS 4 to 3 | 2 (4.9%) | 0 | 3 (7.3%) | 2 (4.9%) | 1 (2.4%) | 2 (7.4%) |
| LI-RADS 5 to 3 | 0 | 0 | 1 (2.4%) | 1 (3.7%) | 0 | 0 |
| LI-RADS 5 to 4 | 2 (4.9%) | 0 | 1 (2.4%) | 1 (3.7%) | 3 (7.3%) | 2 (7.4%) |
